# Supplementary material for: The interactome of intact mitochondria by cross-linking mass spectrometry provides evidence for coexisting respiratory supercomplexes
Source: Mol Cell Proteomics. 2017 Dec 8;17(2):216–32. doi: 10.1074/mcp.RA117.000470 (PMC5795388; doi:10.1074/mcp.RA117.000470)
Supplement: Supplemental Data [file supp_RA117.000470_133922_0_supp_23978_fzffwf.zip › spectra_annotation/mito_spectra_annotation/143-1-4-1-8-1.pdf]

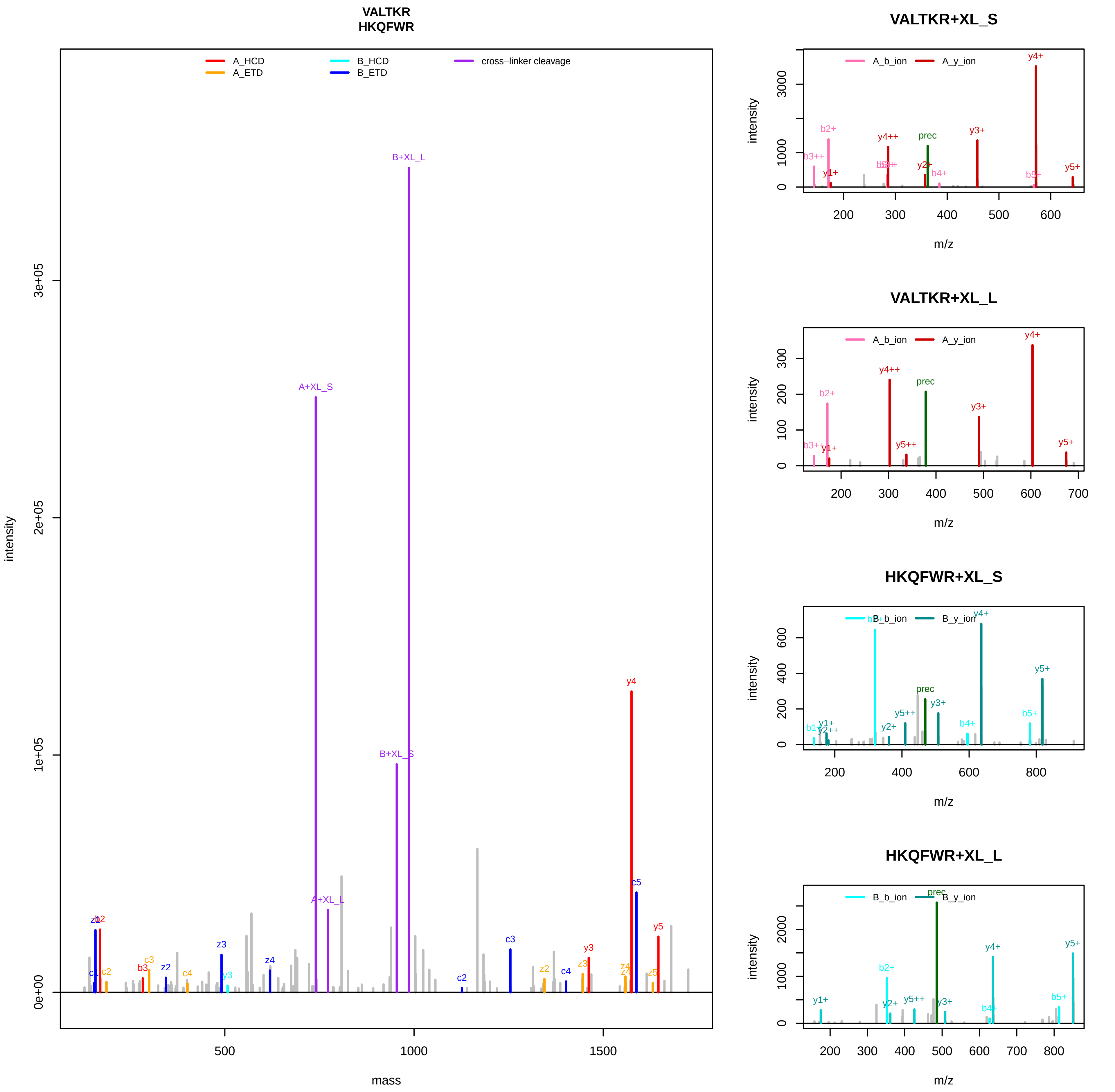

VALTKR
HKQFWR
A_HCD
A_ETD
B_HCD
B_ETD
cross−linker cleavage
B+XL_L
A+XL_S
B+XL_S
A+XL_L
y4
c5
y5
y3
z3
z4
z5
c4
z2
c3
c2
z4
z3
z2
c4
c3
c2
c1
z1
b2
b3
y3
0e+00
1e+05
2e+05
3e+05
500
1000
1500
mass
intensity
VALTKR+XL_S
A_b_ion
A_y_ion
y4+
b2+
y4++
prec
y3+
b3++
y1+
y2+
b4+
b5+
y5+
VALTKR+XL_L
y5++
HKQFWR+XL_S
B_b_ion
B_y_ion
b2+
y1+
y2++
y2+
y5++
y3+
b4+
b5+
b1+
HKQFWR+XL_L
m/z
intensity
